# Choosing a medical specialty: the difference between what students want and what society needs

**DOI:** 10.1186/2045-4015-2-18

**Published:** 2013-05-21

**Authors:** David M Mirvis

**Affiliations:** 1 Department of Public Health, University of Tennessee, Knoxville, USA; 2 Methodist LeBonheur Center for Healthcare Economics, University of Memphis, 5676 Redding Avenue, Memphis, TN 38120, USA

## Abstract

The choice of a specialty by medical students is a complex one that has significant implications for the future supply of physician manpower. The study by Weissman et al. portrays this choice as reflecting the degree of congruence between a student’s needs and values and his or her perception of the characteristics of the various specialties. The existing shortages in the supply of various specialists in Israel may be interpreted as signifying a lack of alignment of student needs and perceptions. This commentary will extend the implications of this work to include the connection between students’ choices and the physician manpower needs of society, and will focus on primary care physician shortages in the United States as but one example of the implications of these relationships.

## 

The report by Weissman and his associates [1] expands a large and, at times, confusing literature seeking to understand how medical students choose their future practice specialty. These efforts are important because graduate medical education is the “spigot controlling the output of the physician workforce pipeline” [2]. This, then, determines the future supply of primary care and specialist practitioners in the community. In this commentary I will extend the implications of this work to include the connection between students’ choices and the physician manpower needs of society. In doing so, I will focus on primary care physician shortages in the United States as but one example of the implications of these relationships.

As described in this report and in the broader literature, the choice of a specialty is a complex [3,4] and important one for the student that has significant implications for the health care and the health of the community. One set of determinants assessed by Weisman et al. [1] reflects student needs and values. These include, among others, desires for a controllable lifestyle, intellectual stimulation, and income.

This first set of determinants interacts with a second set that includes the characteristics of the specialties (e.g., work hours) and the attitudes of the community and other professionals about the specialties (e.g., prestige), as perceived by the students. As portrayed by the market research model used by Weissman et al. [1], a critical factor is the degree of convergence between these two sets of determinants, that is, the degree to which a student’s needs and values correspond with his or her perception of the various specialties.

The relevance of this alignment is shown in the results of Weissman et al. [1] as well in other reports from various nations and regarding a variety of different specialties [5,6]. In the U.S., it may be best exemplified by the relationship between the choice of a high paying subspecialty rather than a lower paying primary care field by students with higher student debt [7]. Studies have also demonstrated that individual factors vary among the various primary care specialties [8] and that similar factors play in the choice of subspecialties among internal medicine residents [9].

The extent of this congruence may then be expected to influence the final choice of students and hence the future supply of physicians. This supply, in turn, may then be assessed in relation to the needs of the community for physician services. Thus, the perceptions of students relates directly, in this market research model, to the adequacy of physician supply to meet community requirements, so that the issues and concerns of students and of the professional and community are inextricably linked.

The shortage of primary care and other physicians suggests that the relationship between the perceptions and preferences of students about primary care and certain specialties is not in balance with societal needs. In the United States, access to primary care physicians is a critical concern. The number of medical students considering primary care training is low and falling, while the demand is high and growing. Between 2001 and 2010, there was a 13.6% increase in all residents but a 6.3% decrease in the number expected to enter primary care [10]. Huang and Finegold [11] recently estimated that 48 million Americans live in areas in which the projected need for primary care physicians is more than 5% above the currently available supply, a shortage that will be aggravated by plans to expand health insurance to over 12 million previously uninsured by the Patient Protection and Affordable Care Act (PPACA) [12]. The shortfall and related limits on access to primary care as compared to other nations [13] have been related to the United State’s low population health rankings [14]. In Israel, Weissman et al. [1] suggest an “existential” shortage in general surgery and anesthesiology as well as in primary care.

Because of its importance, the factors that underlie this imbalance has been the subject of much study – and angst – among health system planners in many nations for many years. The challenge posed in this paper by Weissman et al. [1] is to determine how best to mitigate the imbalances between students perceptions of the specialties and their desires and values. Some potential policy responses seem, perhaps deceptively, straightforward. For example, the PPACA in the United States includes provisions to increase payment rates for designated primary care services in publically funded insurance plans. In the U.S., primary care practitioners can expect to earn $1-3 million less over than career than other physicians [15], and expect their expenses to exceed earning for the first three to five years of practice [16]. Other interventions may improve efficiency and reduce nonclinical workload burdens [17]. And as (or if) the number of primary care providers increases, workload per practitioner may fall.

Others involve the medical education institutions. The role of the medical school experience is supported by the high proportion of students who enter medical school with the intent of practicing primary care who change their plans to the subspecialties later during medical school experience [18]. Although the importance of the clinical years of education have received the most attention, the role of the preclinical, basic science curricular should not be underestimated. Factors such as the organizational culture and commitment to the mission of educating future primary care physicians [4,19] and specific curricular interventions are important [4]. The individual “institutional decisions create a meta-curriculum that frames other components of a medical school” [19]. Others have suggested changes in admission criteria to preferentially select future students predisposed to primary care [20], including those exhibiting altruistic attitudes and a greater sense of social responsibility [4].

Yet other factors and perceptions are even more difficult to mitigate. Approaching the problem of the low prestige of shortage specialties is very important although difficult. This loss of esteem exists both among the public and within the profession, and what students identify as a feeling of low prestige is realistic. In the data collected by Weissman [1], only 7% of Israeli students considered family medicine to be a prestigious career, whereas 78% considered general surgery to be so. Similar results have been reported in, for example, Australia in which general practice ranked eleventh among 15 medical specialties in prestige; general surgery ranked first [21]. To many in the public, the primary care provider’s role is viewed as a bureaucratic one rather than as a professional one – akin to a ticket taker who must punch your ticket before going on the “real doctor”, the specialist. To many in the medical profession, the primary care physician is akin to a jack of all trades and a master of known. As described by Robert Petersdorf, “the generalist specialties suffer from the Rodney Dangerfield syndrome – they get no respect” [22].

Several caveats exist in applying the results from Weissman’s study. First, the connection between self-reported student preferences and actual final decisions is not direct; a large proportion of residents entering primary care training programs later decide to subspecialize. In the U.S., as few as 40% of trainees in primary care actually enter primary care practice [23]. Moreover, a medical student’s specialty preference does not always result in that student undertaking residency training in the preferred specialty. During the internship year the student’s preferences may change. In addition, young physicians’ attempts to obtain residency slots in their preferred specialty are not always successful.

Second, projections about future need are based on speculations about changes in health and health care in the society and about the needs of students. The desires of students vary over times, with an increase in emphasis on controllable lifestyle in recent years [24].

Third, perceptions vary from society to society. For example, in the United States, anesthesia is considered to have a controllable lifestyle and general practice is considered to have an uncontrollable lifestyle [25] – the opposite of that reported among Israeli students [1].

Fourth, determining the need for physicians is difficult so that assessing the real impact of the lack of convergence of students’ perceptions and values is imprecise. Factors such as the gender distribution among physicians and the utilization of advanced practice nurses can influence physician demand, as can the staffing pattern and physical layout of practice sites [26]. As health system analyst Eli Ginzberg concluded “… it may be futile to pose the question whether the nation has too many, too few, or just the right number of physicians a decade or two in the future. All we can hope to do is to address selected facets of the supply problem as they force themselves on to the nation’s agenda. To do more is likely to lead to frustration; to do less is to stockpile problems for the future” [27].

## Authors’ information

David M. Mirvis, MD is an Adjunct Professor in the Department of Public Health, University of Tennessee and a Senior Research Fellow in the Methodist LeBonheur Center for Health Care Economics, The University of Memphis, Memphis, Tennessee USA.

## References

[B1] Weissman C Tandeter H Zisk-Rony RY Weiss YG Elchalal U Avidan A Schroeder JE Israeli medical students’ perceptions of six key medical specialties Israel Journal of Health Policy Researchin press 10.1186/2045-4015-2-19 PMC3668158 23692660

[B2] Schwartz MD The US primary care workforce and graduate medical education policy JAMA 2012 308 2252 10.1001/jama.2012.77034 23212505

[B3] Bland CJ Meuer LN Maldonado G Determinants of primary care specialty choice: a non-statistical meta-analysis Acad Med 1995 70 620 10.1097/00001888-199507000-00013 7612128

[B4] Phillips RL Dodoo MS Petterson S Specialty and Geographic Distribution of the Physician Workforce: What Influences Medical Student and Resident Choices 2009 Washington, DC: The Robert Graham Center

[B5] Lambert T Goldacre R Smith F Goldacre MJ Reasons why doctors choose or reject careers in general practice: national surveys Br J Gen Prac 2012 62 e851 10.3399/bjgp12X659330 PMC3505419 23211266

[B6] Newton DA Grayson MS Thomson LF Money, lifestyle, or values? Why medical students choose subspecialty versus general pediatric careers Clin Pediatr 2012 49 116 10.1177/0009922809350216 20080517

[B7] Rosenblatt RA Andrilla CHA The impact of U.S. medical students’ debt on their choice of primary care careers: an analysis of data from 2001 Medical Student Graduation Questionnaire Acad Med 2005 80 815 10.1097/00001888-200509000-00006 16123459

[B8] Freed GL Dunham KM the Research Advisory Committee of the American Academy of Pediatrics All primary care trainees are not the same: the role of economic factors and career choices Pediatrics 2010 125 573 10.1542/peds.2009-1976 20123774

[B9] West CP Drefahl D Popkave C Kolars JC Internal medicine resident self-report of factors associated with career decisions J Gen Intern Med 2009 24 946 10.1007/s11606-009-1039-0 19551448 PMC2710478

[B10] Jolly P Erikson C Garrison G U.S. graduate medical education and physician specialty choice Acad Med 2013 88 468 10.1097/ACM.0b013e318285199d 23425979

[B11] Huang ES Finegold K Seven million Americans live in areas where the demand for primary care may exceed supply by more than 10 percent Health Aff 2013 32 614 10.1377/hlthaff.2012.0913 23426931

[B12] Ku L Jones K Shin P Bruen B Hayes K The states’ next challenge – securing primary care for expanded Medicaid populations N Engl J Med 2011 364 6 493 10.1056/NEJMp1011623 21268720

[B13] Sandy LG Bodenheimer T Pawlson LG Starfield B The political economy of primary care Health Aff 2009 28 1136 10.1377/hlthaff.28.4.1136 19597213

[B14] Starfield B Shi L Groxer A MAcinko J The effects of specialist supply on populations’ health: assessing the evidence Health Aff Web Exclusives 2005 W5 97 10.1377/hlthaff.w5.97 15769797

[B15] Leigh JP Tancredi D Jerents A Romano P Kravitz RW Lifetime earnings for physicians across specialties Med Care 2012 50 1093 10.1097/MLR.0b013e318268ac0c 22922430

[B16] Palmeri M Pipas C Wadsworth E Zukoff M Economic impact of a primary care career: a harsh reality for medical students and the nation Acad Med 2010 85 1692 10.1097/ACM.0b013e3181f5b754 20881829

[B17] Baron RJ What’s keeping us so busy in primary care? N Engl J Med 2010 362 1636 10.1056/NEJMon0910793 20427812

[B18] Durning SJ Elnicli M Cruess DF Almost internists: analysis of students who considered internal medicine but chose other fields Acad Med 2011 86 194 10.1097/ACM.0b013e3182045ee5 21169784

[B19] Smith SR A recipe for medical schools to produce primary care physicians N Engl J Med 2011 364 496 10.1056/NEJMp1012495 21306236

[B20] Witzburg RA Sondheimer HM Holistic review – shaping the medical profession one applicant at a time N Engl J Med 2013 368 1565 10.1056/NEJMp1300411 23574032

[B21] Creed PA Searle J Rogers ME Medical specialty prestige and lifestyle preferences for medical students Soc Sci Med 2010 71 1084 10.1016/j.socscimed.2010.06.027 20674118

[B22] Petersdorf RG General internal medicine: fad or future? J Gen Intern Med 1989 4 527 10.1007/BF02599554 2585162

[B23] West CP Dupras DM General medicine vs subspecialty career plans among internal medicine residents JAMA 2012 308 2241 10.1001/jama.2012.47535 23212502

[B24] Schwartz MD Durning S Linzer M Hauer KE Changes in medical students’ views of internal medicine careers from 1990 to 2007 Arch Intern Med 2011 171 744 10.1001/archinternmed.2011.139 21518941

[B25] Dorsey SR Jarjoura D Rutecki GW The influence of controllable lifestyle and sex on the specialty choices of graduating U.S. medical students, 1996–2003 Acad Med 2005 80 791 10.1097/00001888-200509000-00002 16123455

[B26] Reinhardt UE Ginzberg E Health manpower planning: the case of physician supply “Health Services Research. Key to Health Policy” 1991 Boston: Harvard University Press

[B27] Ginzberg E Physician supply in 2000 Health Aff 1989 8 84 10.1377/hlthaff.8.2.84 2744698

